# Occupational exposures and associated risk factors among U.S. casino workers: a narrative review

**DOI:** 10.3934/publichealth.2018.4.378

**Published:** 2018-10-10

**Authors:** Jessica Miller Clouser, John C. Flunker, Jennifer E. Swanberg, Gail Betz, Surjeet Baidwan, J. Kathleen Tracy

**Affiliations:** 1 Center for Health Services Research, College of Medicine, University of Kentucky, Lexington, KY, USA; 2 College of Public Health, Department of Epidemiology, University of Kentucky, Lexington, KY, USA; 3 School of Social Work, University of Maryland Baltimore, Baltimore, MD, USA; 4 Department of Epidemiology and Public Health, School of Medicine, University of Maryland Baltimore, Baltimore, MD, USA; 5 Health Sciences and Human Services Library, University of Maryland Baltimore, Baltimore, MD, USA; 6 Coppin State University, 2500 West North Avenue, Baltimore, MD, USA; 7 Maryland Center of Excellence on Problem Gambling, University of Maryland Baltimore, Baltimore, MD, USA; 8 Providence College, Providence, Rhode Island, USA

**Keywords:** casino workers, narrative review, occupational health

## Abstract

We conducted a narrative literature review of U.S. casino occupational health and safety research based on the following inclusion criteria: 1) focused on workers, 2) provided information pertaining to exposures present in the occupational environment (e.g., hazards, stressors, etc.), and 3) pertained to casino, gaming, or gambling workers. Following a multi-step process, a total of 11 articles were identified that related to the occupational health and safety of U.S. casino workers. These articles primarily focused on environmental tobacco smoke (ETS) exposures (n = 7 articles), with the remaining articles related to casino worker risk behaviors (i.e., problem gambling and drinking) (n = 2), and psychosocial stressors (n = 2). Our results demonstrate that the overwhelming consensus in the literature is that ETS leads to high respirable particulate matter (PM2.5), tobacco toxin levels and exposures among gaming employees. Our results also suggest that harassment, low autonomy at work, and unsafe work conditions may be of concern, especially for female workers. We identified major gaps in the casino worker occupational safety literature including a lack of studies that evaluated noise exposure, injury data, ergonomics, psychosocial hazards, or long term respiratory health outcomes related to ETS exposure. Future research regarding the occupational safety and health of U.S. casino workers should address these gaps in the literature.

## 1. Introduction

Casino gambling has become a ubiquitous form of entertainment and revenue for individual states in the United States. As a result, casinos have also become a major employer. In 2015, more than 350,000 people were directly employed by commercial casinos nationwide and 24 states had commercial casinos [1]. That same year, $38.54 billion were spent by consumers at U.S. commercial casinos [1]. Tribal casinos also have a major presence in 28 states, with reported revenue of $29.9 billion in gross gaming revenues in 2015 [1].

The relationship between a casino and the surrounding community holds the potential for tension. Casinos provide a means of employment, a source of tax revenue, and entertainment for local residents as well as tourists. The casino industry serves as major source of revenue to state and local economies as well as nearby businesses. However, the adverse effects of gambling addiction on financial and social outcomes among gambling consumers is well documented [2]–[4], with pathological gambling being associated with increased intimate partner violence [5], criminal behavior, family/marital problems, and declines in physical and mental health [6]. While some research is inconclusive on the connection between casinos and crime in neighboring communities (e.g., [7]), associations have been documented between the introduction of a casino into a community and increased crime [8],[9], increased prevalence of problem and pathological gambling, and demand for government and social services [4]. While the economic impact of creating jobs and stimulating the economy is often a driving force behind the introduction of casinos [4], the benefits of casino gambling must be considered within the context of potential harms. For example, what is the quality of casino jobs and what are the occupational health exposures experienced by employees in a casino environment?

Much of the research on the occupational safety and health of casino or gaming workers has been conducted in foreign settings. Such studies have found that casino workers cited indoor air quality arising from second hand smoke [10]–[12]; ergonomic issues related to standing for long hours, lifting and pushing/pulling money, repetitive strain due to card dealing and poorly designed work stations [10],[12]; stress resulting from job duties, customer relations, and the lights, noise, and pace of a super charged environment [10],[12],[13]; as well as temperature extremes [10] all as routine occupational health hazards that should be priority areas for intervention.

Some stressors experienced by casino workers in the extant research are similar to those confronted by other hospitality workers, such as job insecurity, shift work, and the emotional strain of managing stressful customers while maintaining a calm, friendly demeanor [12],[13]. Other factors, however, are unique to the gaming environment. These include the strain of forming relationships with clients who may suffer significant personal consequences through losses incurred; a sense of role conflict inherent in being tasked both to encourage gambling among patrons while also screening for signs of problem or pathological gambling; and the underlying tension inherent to an environment where patrons are vulnerable to significant losses, potentially resulting in hostilities, blame, and/or verbal or physical aggression [12]–[15].

In addition to physical and psychosocial stressors found in international research, gaming workers are at risk of adopting behaviors to which they are exposed at the workplace, including problem or pathological gambling (e.g., [12],[16]–[18]. A study conducted in Queensland, Australia found that problem gambling rates among gaming venue staff were 9.6 times higher than the general population [18]. Another study of casino workers in Macao found an elevated prevalence of risk behaviors among staff that included smoking cigarettes (24%), playing electronic games (45%), playing electronic games more than 3 hours a day (19%), using addictive substances (12%), gambling (14%), having financial difficulties due to gambling (13%), and having family/interpersonal conflicts due to gambling (15%) [12].

As previously mentioned, much of the research that has assessed the physical and psychological occupational health exposures of casino workers has focused on foreign settings, particularly major gaming hubs such as Queensland, Austria and Macao, China. However, due to labor laws, social policies, and gaming regulations that vary internationally, an assessment of the job conditions and occupational health hazards specific to the U.S. casino gaming environment is warranted. To this end, this narrative literature review seeks to identify the physical and psychosocial occupational health hazards and exposures experienced by U.S. casino gaming workers.

## 2. Methods

### 2.1. Study selection

Studies were initially selected based on the following criteria: 1) focused on workers, 2) provided information pertaining to exposures present in the occupational environment (e.g., hazards, stressors, etc.), and 3) pertained to casino, gaming, or gambling workers. The inclusion criteria were refined after the initial search to include those articles that possessed these three criteria and were also conducted in the U.S.

### 2.2. Search strategy

Search strategies were developed in collaboration with a research librarian and included terms related to the concepts of casino workers and occupational health and safety. PubMed, Scopus, SocINDEX and PsycINFO were searched in March 2017 (see Table 1 for a complete search strategy from PubMed). A combination of keyword and subject term searching was used to maximize relevance and retrieval in each database. No language or publication date restrictions were applied. Grey literature was searched to identify dissertations using the ProQuest Dissertations and Theses database; conference proceedings were identified using Scopus.

**Table 1.** Search terms for literature review.

PubMed		
1	2	3
Gambling (MeSH)	Health (MeSH)	Workplace (MeSH)
Gaming	Safety (MeSH)	Occupation (MeSH)
Casino	Risk (MeSH)	Employment (MeSH)
Casinos	Noise (MeSH)	Job
	Human Engineering (MeSH)	Employee
	Condition	Employees
	Conditions	Worker
	Well being	Workers
	Well-being	Staff
	Wellbeing	Work
	Stressor	
	Stressors	
	Exposure	
	Exposures	
	Exposed	
	Psychosocial	
	Physical	
	Hazard	
	Hazards	
	Hazardous	
	Ergonomic	
	Ergonomics	
	Chemical	
	Musculoskeletal	
	Injury	
	Injuries	
	Injured	
	Injurious	
	Illness	
	Illnesses	
	Danger	
	Particulate	
	Respiratory	

**Figure 1.**
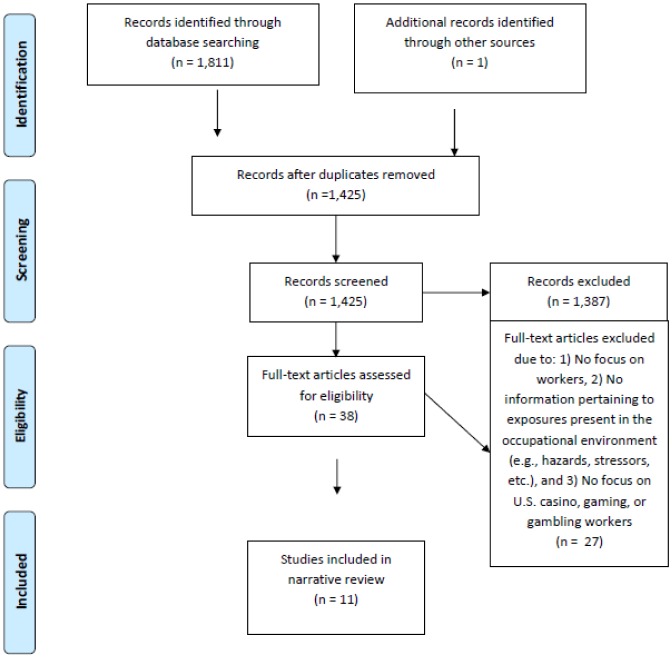
PRISMA flow diagram.

A total of 1,811 articles were retrieved from the initial search, which was reduced to 1,424 after deduplication. An additional article [19] that matched the selection criteria was later identified by the team and added to the list of eligible articles for a total of 1,425. One team member reviewed the title and abstracts of the 1,425 articles and removed those that did not meet the initial criteria, leaving 38 articles. Following the first review, two team members reviewed the title, abstract, and text when necessary, for final determination of inclusion. The second review, which included the additional criteria of being conducted in the U.S., reduced the total number of articles from 38 to 11 (i.e., N = 26 articles were removed). All articles that were excluded from the second review process, in addition to those that were kept, were reviewed by a third team member and any discrepancies in retention and/or elimination were discussed until consensus was reached. The final set of 11 publications represented work from 9 research teams (see Figure 1).

## 3. Results

### 3.1. Literature search results

The 11 articles retained from our literature review fell into three broad categories of casino-related occupational hazards: 1) those pertaining to environmental tobacco smoke (seven publications), 2) those pertaining to casino worker risk behaviors (two publications), and 3) those pertaining to psychosocial stressors (two publications). The specific details of each study can be found in Table 2. Here we summarize the results of these tables by major exposure type.

**Table 2.** Casino worker health research studies in the U.S (n = 11).

First Author (Year)	Risk Factor	Exposure	Study objective	Study Design	Population/sample	Primary results
Achutan (2011)	Environment	ETS^a^	Quantify casino dealers' exposure to ETS.	Personal breathing zone air samples were collected on a convenience sample of 113 casino dealers, including 110 who provided cotinine urine samples each day, pre- and post-shift.	Las Vegas dealers	High level of dealer exposure to ETS and associated constituents of tobacco combustion. Increase in continue throughout shift.
Marin (2011)	Environment	ETS	Compare levels of PM2.5 and cotinine among a sample of casino workers in the San Juan metropolitan area before and after the smoking ban.	Pre and post smoking ban comparisons made via PM2.5 area samples and worker cotinine levels.	San Juan PR casino employees	Smoking ban reduced PM2.5 and saliva cotinine levels.
Repace (2011)	Environment	ETS	Determine impact of ETS on PM2.5 and particulate polycyclic aromatic hydrocarbons (PPAH) in smoking casinos.	Indoor versus outdoor and smoking versus non-smoking casinos/areas PM2.5, PPAH Estimate cardiovascular risks of exposure	NV, CA, DE, NJ, PA	Smoking increases PM2.5 and PPAH in smoking casinos and in non-smoking regions of smoking casinos. Levels of toxins likely to adversely impact respiratory health of workers and patrons.
Repace (2009)	Environment	ETS	Evaluate ETS exposure in terms of respirable suspended particles (RSPs), Particulate polycyclic aromatic hydrocarbons (PPAHs), Carbon dioxide, and measure Cotinine in patron urine.	Compare RSPs, PPAH, Carbon dioxide between indoors and outdoors of smoking casinos. Measure cotinine in patron urine. Measure RSPs from breathing zone of patrons.	PA casino	Smoking increases RSPs and PPAH levels. Cotinine in urine also increases due to smoking.
Trout (1998)	Environment	ETS	Evaluate ETS exposure among casino dealers and supervisors.	Breathing zone sampling and area sampling for nicotine vapors and respirable dust. Pre and post shift cotinine in urine and serum.	Atlantic City, NJ dealers and supervisors	Dealers and supervisors have higher level of ETS exposure than general population.
York (2010)	Environment	ETS	Assess PM2.5 in Nevada smoking casinos Compare the PM2.5 in casino gaming areas to their attached nonsmoking restaurants after enactment of the NCIAA.	PM2.5 sampling in smoking and non-smoking areas of casino. Sampling via personal monitors on patrons.	Las Vegas, NV	PM_2.5_ levels above EPA designated healthy levels in smoking casinos.
Jiang.(2011)	Environment	ETS	Measure PM2.5 concentrations in CA Native American operated smoking casinos. Explore differences due to casino size, separation of smoking/non-smoking areas, and area smoker density.	Comparison among small, medium, and large casinos (by number of slot machines) in terms of PM2.5, smoking barriers, and smoking versus non-smoking regions. Sampling via personal monitors on patrons	CA Native American operated casinos	Elevated PM2.5 due to smoking. Lack of smoking barriers had no impact on PM2.5. Complete separation lessened exposure to outdoor levels while partial separation lessened PM2.5 levels.
Shaffer (1999)	Work behaviors	Problem and patho-logical gambling, Problem drinking, Smoking	Identify prevalence of gambling problems, alcohol problems, and tobacco use among casino employees, as well as associations with depression.	Survey distributed to workers at multiple sites. Assessed Pathological gambling, problem gambling (SOGS), alcohol problems (CAGE), depression, smoking and demographics.	Casino Inc. employees	Casino employees were more prone to pathological gambling, alcohol use, smoking, and depression than general U.S. population, but experience lower risk for problem gambling. Tenure is closely related to pathological gambling.
Shaffer (2002)	Work behaviors	Problem gambling Problem drinking	Prospectively examine casino employees' gambling and alcohol usage.	Prospective cohort study with 3 repeated measures (1 per year) on full-time employees: gambling and drinking screening and depression questionnaire.	Casino Inc. employees	Depression and dissatisfaction with life were related to gambling and drinking problems. Females were more prone to reversing problem drinking to less severe levels than males; no difference in sexes in problem gambling reduction.
Jones (2001)	Psychosocial/physical stressors	Psychosocial/physical exposures	Document experience of women who work as maids, cooks, hostesses, change persons, waitresses, and dealers in casinos in northern Nevada.	Descriptive qualitative study with semi-structured open ended interview	Reno, NV female casino employees	Female casino workers experience a wide array of physical, psychosocial and respiratory exposures; also exposure to risk behaviors, such as drug, alcohol and gambling addictions.
Stedham (1998)	Psychosocial/physical stressors	Sexual harassment	Evaluate the relationship between sexual harassment, job satisfaction, organizational commitment, and turnover.	Questionnaires distributed on-site to assess job satisfaction, organizational commitment, and voluntary turnover.	Reno, NV casino employees	Sexual harassment at similar rates to other industries. Harassed employees were less satisfied with jobs and work itself, less satisfied with supervision, and less committed to the organization. Sexually harassed workers tended to be younger, Caucasian, less experienced in the gaming industry, and work as dealers.

Note: a = environmental tobacco smoke.

### 3.2. Environmental tobacco smoke

The majority of the studies identified in our review (7/11 studies) evaluated environmental tobacco smoke (ETS) exposure in the casino (see Table 2), with all seven ETS studies demonstrating increased particulate levels in smoking casinos. These studies evaluated ETS in the casino through a variety of exposure metrics, including: Respirable particulate matter (PM2.5); respirable dust in worker breathing zone and area sampling (7/7 studies); cotinine in urine, blood, or saliva (4/7 studies); area sampling for aromatic hydrocarbons (3/7 studies); area and breathing zone sampling for nicotine vapor (2/7 studies); and an assortment of ETS-related chemicals (e.g., volatile organic compounds (VOCs), aldehydes, etc.) via breathing zone and area sampling (1/7 studies).

Sampling methods to evaluate worker ETS particulate exposures levels included area sampling and breathing zone sampling (1 study), measured pre- and post-smoking ban reductions in particulate levels (1 study), indoor versus outdoor particulate level comparisons at smoking casinos (3 studies), and comparisons between smoking and non-smoking casino particulate levels (2 studies).

Exposures of specific worker types were isolated to dealers and supervisors. Dealer exposure to nicotine vapor particulate in worker breathing zones was examined by two studies [20],[21], one of which also measured supervisor nicotine vapor exposure [21]. In addition to nicotine vapor, Auchutan et al. [20] examined respirable particles, VOCs, aromatic hydrocarbons, and aldehyde exposure among dealers.

Variation in particulate concentrations by region of the casino was evaluated by three studies. Jiang et al. [19], found the slot machine area of California Native American operated casinos to have particularly high levels of PM2.5 concentrations. Non-smoking areas (gambling and restaurant areas) of smoking casinos were generally found to be lower in particulates than smoking areas, yet higher than outdoors [19],[22]–[24]. Jiang et al. [19] found that non-smoking areas in smoking casinos vary in PM2.5 concentration due, in part, to a variety of physical barriers, with varying efficacy in smoke isolation.

Cotinine was a prevalent ETS worker exposure metric in the literature we reviewed. Four studies ultilized cotinine: Achutan et al. [20] examined cotinine in urine among dealers; Marin and Diaz-Toro [25] measured cotinine in saliva among casino workers of non-specific job type; Trout et al., [21] measured cotinine pre- and post-shift among dealers and supervisors; and Repace [22] assessed ETS worker exposure by proxy from patron urine samples.

### 3.3. Casino worker risk behaviors

Two studies identified by our review [26],[27] focused on casino worker risk behaviors, including problem gambling and problem drinking (see Table 2). These studies concluded that the prevalence of problem gambling and drinking is higher among casino workers than the general population. In fact, one study [26] found that casino workers were more likely to be pathological gamblers (2.1% vs. 1.1%), have a major depressive episode (9.8% vs. 3.7%), have alcohol problems (11.5% vs. 7.4%), and to smoke tobacco (39.3% vs. 25.6%) compared to data for the general U.S. population, though they were less likely to be problem gamblers (1.4% vs. 2.2%). Longer tenure as a casino worker was related to pathological gambling in this study, while working in the gaming part of the casino was not.

The second study, Shaffer and Hall [27], examined changes in gambling and drinking behaviors among casino workers and found little change in gambling addiction over time, yet females exhibited a reduction in drinking habits while males did not. This study did not examine variation in work-related gambling and drinking exposure over the same time period, nor were any occupational factors included in analyses aside from direct involvement with gaming, years working for Casino Inc. and years working in casinos. Consequently, associations between occupational factors and risk behaviors over time are difficult to assess. Both studies used questionnaire assessment of factors related to gambling and drinking problems with assistance and funding from casinos. Although differences in gambling addiction were assessed for gaming versus non-gaming workers, more specific job information (e.g., dealers, slot machine technician, etc.) was not available for either study.

### 3.4. Physical and psychosocial stressors

Stedham and Mitchell [28] found that sexual harassment from co-workers and casino management may be a significant occupational health hazard for female casino workers and that dealers may be most susceptible to harassment (see Table 2). Further, employees that experience work-related sexual harrassment were less satisfied with their jobs, work, and supervisor, as well as less committed to the organization, though they were not more likely to quit. This suggests that these workers may feel obligated to tolerate harassing behavior and perceive it as part of the job. A second study by Jones and Chandler [29] focused exclusively on female casino workers and noted that they may be at risk for unique occupational exposures and subsequent adverse effects. The authors included descriptive results suggesting that female casino workers may be subject to exposures unique to traditionally female casino jobs, such as hotel cleaning and cocktail serving. These jobs may promote exposure to chemicals and awkward, prolonged work postures resulting in respiratory illness and musculoskeletal pain, respectively.

### 3.5. Casino cooperation and research involvement

We also examined casino cooperation and involvement in the research, as casino involvement may potentially impact worker participation rates, worker behavior, and exposure assessment/measurements. Five of the 11 studies in our review explicitly noted voluntary casino management participation in coordinating the research [20],[21],[25]–[28]. Two of the ETS studies reviewed (one continental U.S. casino and one Puerto Rico casino) coordinated sampling with management [20],[25], while another study was based on a NIOSH investigation, which is mandated by OSHA rather than voluntary casino participation [21]. Generally, among the studies reviewed, most PM2.5 area sampling in the continental U.S. was done without the casino's knowledge. The two problem gambling studies we reviewed involved cooperation with management and these studies were funded by the casinos in which they were conducted [26],[27]. One study in our review that investigated sexual harassment involved casino management assistance [28], while our sole qualitative study of occupational safety issues among female casino workers did not involve casino management [29].

## 4. Discussion

Findings from this narrative review reveal that, by far, the most closely studied occupational health issue among U.S. casino workers is environmental tobacco smoke (ETS) or second hand smoke (SHS). In fact, seven of the 11 articles that were captured through the review process focused on ETS. All articles concluded that ETS in casinos increased respirable particulate levels and three of these articles verified ETS exposure with biological methods of exposure assessment. However, our results show that, in general, a more detailed assessment of worker exposure to ETS is needed than what exists in the literature to date. For example, cotinine, used by only three of the seven ETS studies, may provide an important assessment of biological exposure to ETS, in addition to area and breathing zone sampling. Furthermore, the assessment of tobacco incineration constituents, such as polyromantic hydrocarbons, volatile organic compounds, and aldehydes, was conducted by only one study in our review. These ETS constituents, as well as associated worker respiratory health impacts, may help refine assessment of worker exposures to ETS. Finally, none of the studies identified by our review examined worker respiratory health through pulmonary function testing, yet such testing may be useful in detecting undiagnosed lung disease and poor lung function.

The focus of many of our reviewed studies on ETS in the casino work environment may reflect the fact that smoking bans in public places have not been issued on a federal level but are the purview of state and local jurisdictions. In many instances, smoking bans that apply to other public places or workplaces may exclude casinos or gaming venues [30], due to an argument that mandating non-smoking facilities may harm revenues [31]. In fact, among the 36 states that have enacted 100% Smokefree provisions, only 20 apply those restrictions to gaming venues [30]. Further, tribal casinos are not subject to the prevailing smoking laws or regulations in the surrounding areas and may allow or restrict smoking at their discretion. As casinos across the country find ways to circumvent these regulations, whether by creating smoking terraces, cigar lounges, or investing in supplemental ventilation that may enable indoor smoking sections [32], a well-founded knowledge of the effects of these practices on the workers and other patrons is crucial for creating policies that protect workers and the public. Our review of the literature suggests that, in some cases, overflow of second hand smoke from the smoking section to non-smoking sections may occur, illustrating the importance of assessing exposure and associated health impacts among casino workers in various areas of the casino to ensure that casinos' responses to non-smoking regulations are actually effective.

Two of the articles in our review focused on risk behaviors and gambling disorders among casino workers [26],[27]. Though the studies on the topic are few in number, the results mirror findings from international studies that found that casino workers are more likely to abuse alcohol, smoke tobacco, and be pathological gamblers than the general population [12]. The reasons for this heightened prevalence have not been delineated in the U.S., yet it is possible that 1) the gaming industry may attract individuals prone to addictive behaviors and/or 2) being exposed to risk behaviors in the work environment may normalize and increase workers' adoption of them. Thus, exposure to risk behaviors, as an inherent part of casino work, may be considered an occupational exposure to be assessed and mitigated. Future studies may benefit from quantifying worker exposure to problem gambling, variation in problem gambling exposure among casino job types, and subsequent impacts on worker behavior.

Two articles in the review focused on psychosocial stressors and found that women and table game dealers were most impacted by psychosocial stress as an occupational hazard [28],[33]. Findings pertaining to harassment highlighted that for women in particular, casino jobs may be sexualized. While one article found that workers in casinos are no more likely to perceive workplace sexual harassment than other workers, this may be due in part to workers' perception that harassment is expected due to the sexualized nature of their jobs [28]. Despite this, workers who perceived sexual harassment from coworkers were less satisfied with their jobs, believed managers were ineffective and unfair, and did not feel their employer cared about their well-being. They were not, however, more likely to leave their jobs, indicating that these workers may not feel they have other options [28]. It should be noted that this study only probed for harassment initiated by coworkers rather than patrons, which is another potential source of harassment that workers may face. Another U.S.-based study [33], that did not meet our inclusion requirements described three women's experiences of working in the casino industry; all mentioned patron and manager harassment, mandatory work attire that was highly sexualized, and sometimes painful (e.g., tall high heels for servers walking long distances and balancing heavy trays). These studies suggest that future research on casino workers should include sexual harassment measures from both coworkers and clients, quantify the level of harassment experienced from each source, and examine resultant worker effects from each source.

Although ergonomics and noise exposure are commonly documented in occupational safety research in other industries, no U.S. studies were found that assessed these hazards among U.S, casino workers. Evidence from international research suggests that these hazards are problematic for worker health in the casino environment [10]. Furthermore, gaming workers from other countries have cited exposure to poor ergonomics; chemical hazards (e.g., cleaning products and coin dust); and biological hazards through constant interaction with clients, temperature extremes, noisy environments, flashing lights, and poor air quality [10]. Also, casino workers in other countries have complained of pain in the lower back, shoulder, joint, neck and head, hearing loss; eye strain; respiratory and reproductive issues; and ill-health and injuries[10]. Only one study was found that assessed physical symptoms experienced by workers, which found that women casino workers experienced back strain, sore feet, and knee problems [29]. No studies were found that quantified or described common injuries (e.g., nature of injury or body part affected) or their risk factors. Further more, no studies examined the impact of psychosocial stressors on worker health such as unpredictable, unstable, or long work hours.These missing exposures and related injuries are likely present in U.S. casinos and further study may help to reveal potential worker exposures and health impacts.

The findings from this review indicate that an assessment of U.S. casino job quality is worthy of investigatation, as job quality factors may impact worker exposure to occupational hazards. Other research on job satisfaction of casino dealers has revealed that characteristics of the work environment such as distrustful supervisor relationships, a high supervisor-to-employee ratio, low job security, and low autonomy conspired to create low job satisfaction [34]. In fact, 49% of sampled casino dealers strongly agreed that they'd rather be working outside of the casino industry and 53% strongly agreed that they never knew when they would be fired [34]. Workers in lower income communities may be vulnerable to adverse effects from job hazards as they may feel they have few job options and are willing to take on more risky and less desirable jobs. Future research must also determine if these casino jobs add true value to the communities in which they are situated. For example, the National Opinion Research Center [4] found that when casinos entered a community, per capita income tended to stay the same despite the increased jobs brought by the casino. This finding indicated that the jobs brought to the community were not necessarily better and therefore left no improvement in the standard of living.

Access to the casino workforce seems to be the key issue in future studies of casino worker health and safety. Many studies in our review evaluated respiratory hazards discreetly in casinos, while few involved casino management. Both studies that looked at problem gambling among casino workers involved industry support [26],[27]. As over half of the studies in our review did not work with casinos to perform research this may suggest that casinos are reluctant to work with researchers. Because management cooperation is crucial to worker access and in-depth exposure assessment, bridging this gap of trust—e.g., by demonstrating the financial benefit of promoting worker health and safety—may be the crux of future research.

Our review demonstrates the need for additional studies that focus on female casino workers. Many women in the casino workforce are non-English speaking immigrant Latinos, a group with a heightened rate of occupational injury [35]. As such, female casino workers may be at risk for exposure and injury while on the job. Furthermore, many casino jobs are dominated by females, such as beverage servers and cleaning staff, which may have unique hazards. Our results show, in particular, that casino hotel cleaning staff may be subject to fast-paced schedules with limited breaks, low pay, and tasks that may have an adverse impact on musculoskeletal and respiratory health. Furthermore, beverage servers may be subject to harassment from both patrons and casino co-workers and may experience musculoskeletal issues related to mandates to wear high heel shoes during long shifts with constant standing and walking [33].

Findings from this review also indicate that job type may impact the risk of worker exposure to specific casino related hazards. Two ETS studies in our review [20],[21] examined ETS and related PM2.5 exposure among dealers and/or supervisors, concluding that ETS exposures may be especially high among dealers. Furthermore, the study we reviewed pertaining to sexual harassment from co-workers [28] revealed that female dealers may be most susceptible. In addition, the qualitative study we reviewed [29] suggested female cleaning workers may experience job-specific hazards unique to cleaning. However, the two studies we reviewed on problem gambling [26],[27] yielded no associations between workers directly involved with gaming and problem gambling. These findings demonstrate a need for further identification and quantification of job-specific exposures and associated worker effects.

### 4.1. Limitations and summary

This study reports a narrative review of articles retained by our search, which, due to a non-statistical approach to results summary, may make our findings subject to bias. Furthermore, it may be possible that misleading titles and abstracts may have led to missed articles that were relevant. However, the number of missed or eliminated relevant articles is likely minimal, as subsequent topic searches revealed only one additional article. We found a surprising paucity of articles related to the occupational safety of U.S. casino workers, leading to a relatively small number of reviewed articles and a limited number of articles per occupational hazard. This lack of extant literature underscores the importantce future studies examining the occupational health and safety of U.S. casino workers. Although our literature search did reveal a number of articles about non-U.S. casino workers, the probable differences in safety climate, work organization, occupational health and safety regulations, and occupational hazards experienced by non-U.S. casino workers made inclusion in our study difficult to justify. Nonetheless, future studies comparing U.S. to non-U.S. casino worker health and safety may provide helpful information to improve casino worker health and safety worldwide.

Despite the limitations of this study, our narrative review of occupational hazards experienced by U.S. casino workers revealed a substantial gap in knowledge about the full spectrum of occupational hazards and exposures experienced by this worker group. Additional research is needed in order to systematically characterize occupational hazards, assess exposure levels, and assess the resultant health impacts on U.S. casino workers. Indeed, prominent gaps in the U.S. literature on the topic include factors related to noise exposure and its subsequent health impacts, the prevalence of injuries and related occupational factors, the prevalence of and factors related to musculoskeletal issues/disorders, and an assessment of the quality of U.S. casino jobs and how their quality may influence worker health. Research on casino worker risk behaviors that may be motivated by aspects of the work environment (e.g., harassment) have been touched upon by a few researchers but additional work remains to truly understand their scope, antecedents, and health impacts. The U.S. casino industry remains relatively under-investigated in terms of occupational exposures and worker health issues. Yet, with cooperation between the industry and researchers, casino worker health and safety may be better understood and improved.
